# A clinical audit of anatomical side marker use in a paediatric medical imaging department

**DOI:** 10.1002/jmrs.176

**Published:** 2016-05-25

**Authors:** Kate Barry, Saravana Kumar, Rebecca Linke, Emma Dawes

**Affiliations:** ^1^ SA Medical ImagingRoyal Adelaide HospitalAdelaideSouth AustraliaAustralia; ^2^ International Centre for Allied Health Evidence (iCAHE)City East Campus, University of South AustraliaAdelaideSouth AustraliaAustralia; ^3^ SA Medical ImagingWomen's and Children's HospitalNorth AdelaideSouth AustraliaAustralia; ^4^ Queen Elizabeth HospitalAdelaideSouth AustraliaAustralia

**Keywords:** Lead marker, medical imaging, patient safety, practice guidelines, radiography, side marker

## Abstract

**Introduction:**

The gold standard in general radiography is to place a radiopaque anatomical side marker in the field of view for each radiographic image prior to exposure. The advent of digital radiography has allowed for anatomical side markers to be digitally added to films as part of post‐processing. The aim of this audit was to identify whether general X‐ray images performed in a tertiary Women's and Children's Hospital were being appropriately annotated with a definitive side marker, and to identify factors that may contribute to inappropriately labelled images.

**Methods:**

Four hundred images from 201 patients’ examinations occurring within a randomly selected time period were assessed to ascertain whether radiographic anatomical side markers were visible when images were viewed via the hospitals main viewing platform. The audit occurred in January 2014. The scope included both mobile and in‐department general X‐ray examinations, with the patient age range extending from 1 day to 18 years.

**Results:**

Of the 400 images evaluated, 88 (22%) were found to have a lead marker that matched the anatomy being imaged within the primary beam; 289 (72.3%) images contained a correct digital marker inserted as part of the post‐processing of the image. In total, 377 (94.2%) images were appropriately marked. Of the 23 (5.8%) images not marked correctly, 22 images had no marker and 1 was incorrectly marked with a digital marker. There was a noticeable relationship between absent anatomical markers and chest X‐rays performed outside of the medical imaging department.

**Conclusions:**

While it is encouraging that the majority of the images assessed were correctly annotated, with only a small number of missing markers, there are opportunities for further improvement. The audit findings suggest that reduced access to lead markers influences marker use. Strategies that may improve compliance at an individual level include distribution of personalised anatomical side markers, and targeted staff education sessions. At a department level, regular audits and monitoring should be encouraged.

## Introduction

It is globally accepted best practice that all radiographic images must display a correct anatomical side marker.^1^, ^2^, ^3^, ^4^, ^5^, ^6^, ^7^ Anatomical side markers are defined as annotations of ‘right’ or ‘left’ on the image.^3^ In their professional practice guidelines, the Australian Institute of Radiography (AIR) state that to be a competent radiographer one must ‘verify that radiographic markers are present on each image, and that they are accurate’.^1^

Best practice has always been to apply radiopaque anatomical side markers in the primary beam pre‐exposure.^3^, ^4^ Recent technological innovations and change within radiology processes have had far‐reaching implications. A significant recent development has been the introduction of digital radiography in medical imaging. As part of its increased functionality, digital radiography allows post‐processing annotation of images, which enables radiographers to add digital markers to images post hoc. Rather than increasing compliance, however, the addition of digital markers has been found to increase the incidence of incorrect or missing anatomical side markers.^8^

Patient safety and correct anatomical side identification are imperative to the delivery of quality medical imaging services.^1^, ^2^, ^5^, ^6^, ^7^ All radiographic images must carry unambiguous anatomical side markers (right or left).^2^, ^3^, ^5^, ^6^ The placement of anatomical side markers in both the digital and pre‐digital technology era are operator dependent and therefore subject to human errors. Incorrectly marked films can have serious patient safety implications contributing, for example, to wrong site surgery.^7^, ^8^, ^9^, ^10^

In the case study by Finnbogason, Bremmer and Ringertz (2002), the circumstances surrounding two cases in which radiographic side markers were missing from mobile chest X‐rays (CXR) taken in a neonatal intensive care unit (NICU) were discussed.^10^ The study concluded that the lack of anatomical side markers, complicated pathologies and critical condition of these patients led to the insertion of a chest drain on the incorrect side. One case proved to be fatal for the patient; the other experienced no ill effects once the chest drain had been re‐inserted into the correct side.

**Figure 1:**
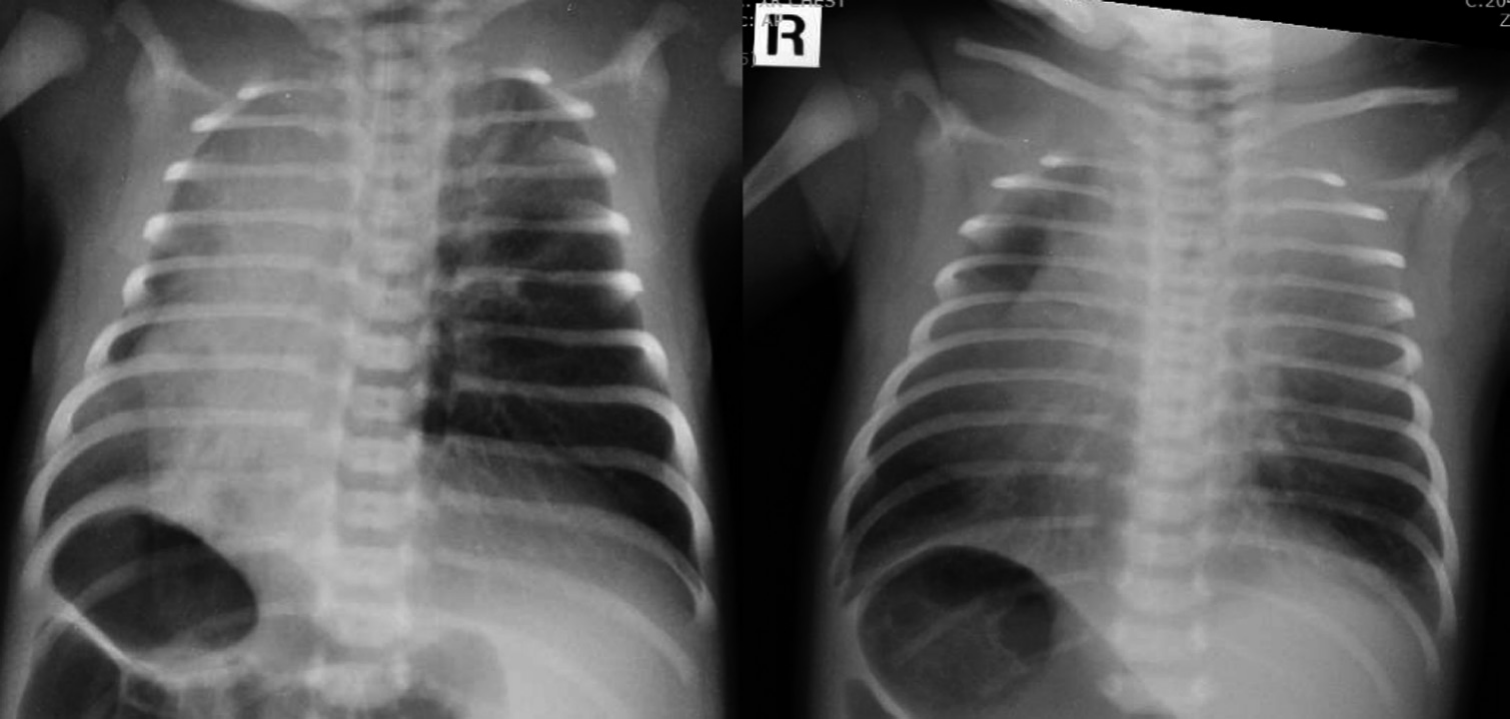
Chest X‐rays taken in the neonatal intensive care unit, the first is lacking lead or digital markers and has the potential to confuse treating clinicians. The repeated image shows a lead marker on the correct side. The patient has situs inversus.

Figure 1 taken from an examination performed at the Women's and Children's Hospital, highlights the importance of accurate use of anatomical side markers. In this case, the patient being imaged had a congenital anomaly known as situs inversus, in which the heart and other organs are transposed through the sagittal plane to lie on the opposite side from the usual. Unless clearly marked by anatomical side markers, an image (Fig. 1) in which the cardiac shadow and gastric bubble are visible on the right side of the chest (rather than the more usual left) has the potential to be incorrectly interpreted by the radiographer or treating clinicians.

Despite the integration of new technologies into local practice at the Women's and Children's Hospital, no systematic reviews had been performed within the medical imaging department to verify that the best practice standards of image annotation were being met. Anecdotal evidence from within the department suggested that there were instances when lead markers were not appropriately used. The aim of this descriptive case note audit was to establish the proportion of images emerging from this department that met best practice standards of image annotation.

Images were evaluated to determine:

- The % of images on which an unambiguous anatomical lead side marker was present
- The % of images on which a digital marker had been placed during post‐processing
- The % of images that had an incorrect or absent anatomical marker.

And to assess whether a relationship existed between:

- Patient age and marker type (lead or digital)
- Acquisition location (mobile or in department) and marker type (lead or digital)
- Anatomical area of image, projection and marker type (lead or digital).

## Methods

### Design

This clinical audit utilised a descriptive research approach to investigate the phenomenon of interest (i.e. the use of anatomical side markers). While descriptive research cannot establish causality, it is a useful tool to describe the extent of an issue, and can guide further research. Descriptive clinical audits are common in healthcare where current practices are compared to accepted best practice standards. Approximately 1 month prior to data collection, the lead researcher conducted a pilot audit of 10 images to establish the utility and accuracy of the audit tool, and ensure effective capture of all relevant data.

### Ethical considerations

Ethics approval was sought and granted by the Women's and Children's Health Network Human Research Ethics Committee prior to data collection. All data extracted and examined for the purpose of this audit had patient‐identifying information removed prior to publication to preserve patient confidentiality.

### Sample size

Power analysis and sample size calculator indicated a sample size of 377 was required to attain statistically significant data.^11^ The sample size was increased to 400 in order to power up the audit. Sample size was established by using a sample size calculator with an acceptable margin of error of 5%, a confidence level of 95%, a population of 20,000 and a response distribution of 50%. In order to obtain an estimate figure for the population the annual statistics for the medical imaging department were reviewed. Data showed that of the approximately 50,000 medical imaging examinations that were performed in the department per annum, 20,000 were general X‐ray examinations. The remaining 30,000 studies consisted of medical resonance imaging, ultrasound, computed tomography and nuclear medicine examinations, which were outside of the scope of this audit. As most patients require more than one image per examination, data were extracted from 300 patient examinations in total, but data from only 201 patients was required to meet the target of 400 images. Patient data were extracted in time order of presentation to the department.

### Inclusion/Exclusion criteria

Data were collected via Radiology Information System (RIS), the data collection system being utilised within the medical imaging department at the time of the audit. Data were extracted by the office administration manager for 300 patients aged between 0 and 18 years old who presented for general X‐ray examinations between 09:00 am on January 22, 2014 until 12:00 on January 24, 2014. Exclusion criteria for general examinations included orthopantomogram and cephalometric studies as equipment used to perform these studies utilised fixed markers. Theatre cases were also excluded as local procedure is not to place lead markers on image intensifiers due to infection control risks in the theatre setting. All other general X‐ray examinations, including mobile examinations performed externally to the department, were included in data collection.

### Procedure

The main viewing platform for clinicians in this hospital is OACIS (Open Architecture Clinical Information System), which has some features of a mini‐PACS (Picture Archive and Communications System). The majority of images are received as DICOM (Digital Imaging and Communications in Medicine) images; however some images are scanned in from film. OACIS access is available to all clinicians, and is installed or available on every PC within the hospital. At the time of audit there was no full radiology PACS in the hospital, and hard copy films were still printed and filed. Few clinicians refer to hard copy films as access to images is much faster via OACIS, and digital access mitigates the risk of lost or damaged films. The exception is the orthopaedic outpatients department who continue to view and template from hard copy images, and the NICU who utilise both hard copy films, and OACIS. The images assessed for the purpose of this audit were all viewed on OACIS, replicating the experience of the majority of referring clinicians.

### Data collection and analysis

The office administration manager was asked to randomly select a 48‐hour period (limited to January 2014), and electronically extract data from RIS to generate a list of all general X‐ray examinations performed in this time period. These data were supplied to the lead author in a spreadsheet via e‐mail. The lead author was the sole data collector and performed the analysis. The spreadsheet listed all general X‐ray examinations performed, and those that met exclusion criteria were manually deleted from the spreadsheet. Data analysis and recording involved using this list to view and analyse images via OACIS, and manually completing an audit table tool from the images viewed. The audit tool contained columns to identify patient age, anatomy being imaged, presence of anatomical side marker, type of anatomical side marker and location of X‐ray. The audit tool was numbered so that audit cut off occurred at 400 images.

All images viewed during the data analysis contained patient identifying information. In order to preserve patient confidentiality this information was not recorded on the audit tool. During data analysis a separate list was compiled of any images noted to have missing or incorrect markers. This list was later used to amend images according to departmental protocol.

A total of 201 examinations were required to yield 400 radiographic images. Of these examinations, 178 occurred within the Medical Imaging Department and 23 were performed outside of the department in wards such as Paediatric Intensive Care Unit, NICU or in the emergency department.

## Results

The gold standard for annotating radiographic images is to include an unambiguous anatomical side marker in the primary beam prior to exposure. This occurred in 88 of the 400 images. A digital marker was present in 289 of 400 images. Eighteen images had both lead and digital annotation. Three hundred seventy‐seven of 400 images were marked appropriately, meaning 23 or 5.8% of images included in the audit had an incorrect or absent anatomical side marker.

Table 1 provides a breakdown of missing or incorrect markers and the relationship with mobile or in‐department imaging. Of the 23 images not marked appropriately, 22 were found to be missing a definitive anatomical side marker (lead or digital) and 1 was incorrectly marked. The incorrectly marked image was amended both on the hard copy and on the digital file accessed by OACIS, and the referring team informed.

**Table 1:** The breakdown of marker compliance for general radiographic examinations

	Lead marker present	Digital marker present	Both markers present	Incorrect or absent marker	Image marked appropriately
Number of images	88	289	18	23	377
Total number of images audited	*N* = 400	*N* = 400	*N* = 400	*N* = 400	*N* = 400
Frequency (%)	22	72.3	4.5	5.8	94.3

In a further breakdown of the data, of the 22 images missing a side marker in Table 1, 12 of these occurred on images taken out of the medical imaging department. Over half (52.2%) of the mobile examinations during the audit lacked anatomical markers. Fewer occurred in department examinations, 10 images from 174 (5.8%) patients lacked anatomical side markers.

In the course of this audit a total of 56 from 400 images were chest x‐rays (CXR). Other audit findings demonstrate that, of the 56 chest X‐ray images attained over the course of the audit, only 3 (5.4%) had a lead marker present, while 42 (75%) had a digital marker present and 11 (19.6%) were found to have no definitive side marker.

The audit also demonstrated that chest X‐rays are the most common mobile X‐ray examination performed at this hospital. The data collected for this audit revealed that of 23 mobile X‐rays, 20 were chest X‐rays. The remaining three were abdominal X‐rays.

The incidence of marker use in relation to patient age is provided in Table 2; of note is the finding that babies and toddlers (0–3) accounted for nearly two thirds of missing markers (59.1%).

**Table 2:** Patient age and the incidence of absent markers

Patient age groups (years)	Number of images with absent markers	%
0–3.	13	59.1
3–6.	2	9.1
6–9.	1	4.5
9–12.	0	0
12–15.	3	13.6
15–18.	3	13.6
Total number of images with missing markers	22	

## Discussion

The aim of this audit was to identify the proportion of general X‐ray images that were appropriately marked with a definitive anatomical side marker. The findings from this audit indicate that of the 400 images reviewed, 23 were incorrectly marked. While nearly 95% of the images were appropriately marked, even the small number of incorrectly marked images raises the potential for adverse incidents. Given the increasing focus on quality and safety in healthcare, any potential threats to patient safety, however minor they seem, should be minimised where possible.

Chest X‐rays are one of the most frequently occurring examinations in any medical imaging department. It is imperative that chest X‐rays are correctly marked by the use of anatomical side markers because conditions such as dextrocardia, situs inversus, or pneumothorax can cause anatomical anomalies that may result in wrong side treatment and potentially harmful consequences for the patient.

The most alarming finding was that over 50% of mobile X‐rays were missing a lead anatomical side marker, which required immediate attention and change in practice. The patient population requiring mobile X‐rays are the sickest in the hospital and most likely to have complicating pathologies. It is likely that difficulty in placing the markers (due to patient size, medical attachments, infection control practices and compliance limits) may act as a barrier to marker placement. Finally, the age of the patient undergoing examination also appeared to influence the use of markers. Furthermore, patients in all age groups from 0 to 18 years, with the exception of those aged 9–12 years, had images missing markers. This indicates that problem is not confined to the very young, and is an area that requires vigilance and ongoing monitoring.

The findings from this clinical audit identified numerous opportunities to improve practice. Achieving change in clinical practice is complex and requires a systematic and planned approach. In response to this audit a range of strategies were implemented and communicated to the Women's and Children's medical imaging staff. First and foremost education about the importance of correctly annotating images is a crucial starting point for empowering staff and increasing marker compliance.^12^ Furthermore, it is necessary to ensure that staff have the required knowledge and technical skills to consistently annotate images with a definitive anatomical side marker. In addition to insufficient education, lack of access to lead anatomical side markers may be a contributing factor to staff compliance. In response, all radiographers in this medical imaging department have been individually provided with two sets of lead anatomical side markers. The introduction of two perspex boards containing spare sets of individually labelled anatomical side markers for every radiographer in the department have further acted to increase ergonomic access to markers. With regard to mobile X‐rays, a spare set of lead anatomical side markers without identifying initials has been attached to each mobile X‐ray machine in the hospital.

The arrival of full radiology PACS at the institution audited was imminent at the time of audit. As such, the value of adding a more effective ‘double checking’ process to current practices was seen to be limited; any new processes were likely to be rendered obsolete by the new operating system. In the light of this, it was recommended that, as a part of the training and education for the transition to PACS, systematic checking for the presence of accurate left and right annotations be included as a routine part of the patient identity data verification process. In this way, any changes required could be implemented rapidly, with notification made to the PACS manager to facilitate immediate deletion of incorrect images. As a contingency system for errors noted outside of office hours, it is advised that a standardised form indicating the error and required amendments be developed, and that radiographers scan completed forms into the system to be added to the patients file.

Radiologist reporting of X‐rays is the final step before results are released to referrers. Currently there is no specific pathway for notification should the radiologist notice that an image is incorrectly labelled. A strategy to address this deficiency would be an approach whereby any radiologist who identifies an absent or incorrect marker is required to liaise directly with the senior radiographer to resolve this and make suitable amendments. The radiologist can, and should, provide an additional set of eyes, and ensure the images are not disseminated prior to all checks being complete. None of the missing or incorrect markers discovered in the audit process were noted or commented on by the reporting radiologist in their written report.

The importance of correct radiographic anatomical side markers should not be underestimated and the most reliable way to ensure this occurs is for the radiographer to apply a lead anatomical side marker pre‐exposure in every instance. The patient demographic of the hospital audited does not allow this as easily as an adult institution might as it is heavily weighted in the age range of 0–10 years. When performing X‐ray examinations on patients of this age, the focus of the radiographer is often on speed and patient distraction, and the extra few seconds required to place an anatomical side marker may be overlooked, with the preference being to obtain diagnostic images. To counteract this, radiographers should work as a team to confirm the presence of correct markers on each image as thoroughly as they perform checks for patient identification and examination verification.

Despite the opportunities for adverse events due to incorrect or missing anatomical side markers, there is little research in this area. While there are studies regarding adverse events in hospitals this research does not extend to radiographic anatomical side makers.^8^, ^13^ It is likely that incorrect or missing radiographic markers can and do contribute to adverse events, and that correctly applied anatomical side markers will reduce potential risk. With the implementation of full radiology PACS, and with ongoing educational strategies, it is hoped that the existing error rate will decrease and marker compliance increase to 100%.

## Limitations and Future Research

Several limitations to this audit have been identified and are outlined below. A descriptive clinical audit does not establish causality and as such, cannot answer how, where and why. The risk of performance bias while conducting this audit is noteworthy, one of the authors of this paper was also one of the radiographers whose images were assessed.^14^, ^15^ To reduce bias, although all members of the radiographic team were aware that an audit was to be undertaken, no one (including the author) was aware as to when the audit data would be extracted and which random day(s) would be selected by the office manager. Furthermore, the subject of the audit was discussed within the department for 6 months prior to the date of audit, effectively undermining any short‐term changes in behaviour as a result of observation of practice.

Current practice within this medical imaging department at the time of audit was to use direct capture digital radiography for studies performed within the department, and computed radiography for mobile imaging. One of the drawbacks of the computed radiography system used at the hospital audited, was that during transfer to the OACIS viewing platform images were cropped to include only the field of view. This meant that any post‐processing annotative markers placed outside the field of view were not able to be seen via OACIS. Conversely, all annotative markers, whether placed inside and outside of the field of view were visible on the hard copy film. Staff had been cautioned about this disparity via e‐mail, verbally, and via signs in the image processing area, but despite this there was anecdotal instances where images appeared on OACIS to be missing a side marker even though the hard copy had correct markers present.

One of the limitations of this audit was that it was confined to a single site. It would be ideal to compare the results of this clinical audit with those of other medical imaging departments within the state, in order to allow benchmarking and identification of an error rate. Another limitation noted was the use of OACIS to audit anatomical marker compliance. An obvious limitation is that although it is the preferred viewing platform for many practitioners, it is not the sole option. Many radiographers perform a ‘second check’ for correct anatomical side markers during the process of assessing whether hard copy images are ready for reporting. This check can lead to the detection of missing or incorrect anatomical side markers, allowing the radiographer to correct the error manually through placement of adhesive labels denoting left and right. This practice, however, does not alter the images on OACIS. As such it is possible that some of films taken without accurate markers and uploaded to OACIS may actually have had the anomaly noted, and corrected, on the hard copy prior to distribution of the hard copy films.

## Conclusion

This audit contributes to an emerging field that recognises the importance of anatomical side markers on radiographic images, and highlights the need to audit departmental practice, particularly as the transition is made to the era of digital radiography. Furthermore, it has provided the hospital audited with new information regarding radiographer compliance and the application of anatomical side markers, allowing for the formation of recommendations for future improvement.

The findings from this audit indicate that, in the department being audited, radiographers are more likely to utilise post‐processing digital anatomical side markers, than use pre‐exposure anatomical side markers, despite the latter being considered best practice. While it is encouraging that the majority of the images assessed were correctly annotated, with only a small number (5.8%) missing markers, there are opportunities for further improvement. The audit identifies two factors that may correlate with missing and incorrect markers. These are younger patient age, and imaging being performed elsewhere in the hospital at sites external to the medical imaging department.

An unambiguous anatomical side marker representing the correct anatomical side on 100% of images is the accepted best practice, and it is reasonable that any organisation should strive to meet this target. Two strategies likely to improve compliance in this area are increasing education for staff, and improving access to lead anatomical side markers. To ensure lasting change, time and resource efficient strategies to address these issues must be integrated into routine work as part of the standardised and transparent departmental practices. Some of the strategies outlined above have been successfully trialled within the medical imaging department. As implementing and sustaining change in clinical practice is a long‐term proposition, ongoing education and engagement with radiographers and regular monitoring will be required.

## Conflict of Interest

The authors declare no conflict of interest.

## Supporting information


**Appendix I.** A section of the audit tool used to compile data.

## References

[1] Australian Institute of Radiography . Guidelines for professional conduct for radiographers, radiation therapists and sonographers. [pdf on the Internet] Australian Institute of Radiography. [updated 2013 June 25; cited 2014 April 10] Available from: http://www.air.asn.au/cms_files/05_Accreditation/Professional_practice_standards_finaldraft_180913.pdf.

[2] Frank E , Long B , Smith B . Merrill's Atlas of Radiographic Positioning and Procedures, 12th edn. Mosby, Portland, 2012.

[3] Best Practices in Digital Radiography . White paper [pdf on the internet]. American Society of Radiologic Technologists. [updated 2013 July 9; cited 2014 April 23] Available from: http://www.asrt.org/docs/whitepapers/asrt12_bstpracdigradwhp_final.pdf.

[4] Platt J , Strudwick R . The application of anatomical side markers during abdominal and IVU examinations: An investigation of practice prior to and post‐installation of computed radiography. Radiography 2009; 15: 292–9.

[5] Brennan TA , Leape LL , Laird NM , et al. Incidence of adverse events and negligence in hospitalized patients. Results of the Harvard Medical Practice Study I. N Engl J Med 1991; 324: 370–6. 1987460 10.1056/NEJM199102073240604

[6] Holden JD . Hawthorne effects and research into professional practice. J Eval Clin Pract 2001; 7: 65–70. 11240840 10.1046/j.1365-2753.2001.00280.x

[7] Leonard K , Masatu MC . Outpatient process quality evaluation and the Hawthorne Effect. Soc Sci Med 2006; 63: 2330–40. 16887245 10.1016/j.socscimed.2006.06.003

[8] Australian radiation protection and nuclear safety agency, code of practice: radiation protection in the medical applications of ionizing radiation. [pdf on the Internet] [updated 2013 June 25; cited 2014 April 11] Radiation Protection Series Publication No. 14. Available from: http://www.air.asn.au/cms_files/10_Publications/policies_guidelines/pps_air_dec2013.pdf.

[9] Titley AG , Cosson P . Radiographer use of anatomical side markers and the latent conditions affecting their use in practice. Radiography 2014; 20: 42–7.

[10] Meinberg EG , Stern PJ . Incidence of wrong‐site surgery among hand surgeons. J Bone Joint Surg Am 2003; 85: 193–7. 12571293 10.2106/00004623-200302000-00002

[11] Finnbogason T , Bremmer S , Ringertz H . Side markings of the neonatal chest X‐ray: Two legal cases of pneumothorax side mix up. Eur Radiol 2002; 12: 938–41. 11960251 10.1007/s003300101067

[12] Institute for healthcare improvement [case study on the internet]. IHI Open School [updated 2013 September 13; cited 2014 April 24] Available from: http://www.ihi.org/education/ihiopenschool/resources/Pages/Activities/AHRQCaseStudyXRayFlip.aspx

[13] Aakre KT , Johnson CD . Plain‐radiographic image labelling: Process to improve clinical outcomes. J Am Coll Radiol 2006; 3: 949–53. 17412207 10.1016/j.jacr.2006.07.005

[14] Sample size calculator [page on Internet] Raosoft Inc. [updated 2013 November 26; cited 2013 December 12]. Available from: http://www.raosoft.com/samplesize.html.

[15] Croskerry P . The importance of cognitive errors in diagnosis and strategies to minimize them. Acad Med 2003; 78: 775–80. 12915363 10.1097/00001888-200308000-00003

