# Supplementary material for: A clinical audit of anatomical side marker use in a paediatric medical imaging department
Source: J Med Radiat Sci. 2016 May 25;63(3):148–54. doi: 10.1002/jmrs.176 (PMC5016612; doi:10.1002/jmrs.176)
Supplement: Supplementary file 1 — Appendix I. A section of the audit tool used to compile data. [file JMRS-63-148-s001.docx]

**APPENDIX I**

A section of the audit tool used to compile data.

| **Audit Tool** |  |  |  |  |  |  |  |  |  |
| --- | --- | --- | --- | --- | --- | --- | --- | --- | --- |
| **WCH** |  |  |  |  |  |  |  |  |  |
|  |  |  |  |  |  |  |  | **CXR/AXR/SXR** |  |
|  |  | Marker | Lead marker | Digital & Lead | Digital | Is marker(s) |  | CR/DR |  |
| Exam Number | Image Number | Present | visualized | Markers Present | Marker Only | Correct? |  | X-ray Type mobile/in dept | Patient Age |
|  | 1 |  |  |  |  |  |  |  |  |
|  | 2 |  |  |  |  |  |  |  |  |
|  | 3 |  |  |  |  |  |  |  |  |
|  | 4 |  |  |  |  |  |  |  |  |
|  | 5 |  |  |  |  |  |  |  |  |
|  | 6 |  |  |  |  |  |  |  |  |
|  | 7 |  |  |  |  |  |  |  |  |
|  | 8 |  |  |  |  |  |  |  |  |
|  | 9 |  |  |  |  |  |  |  |  |
|  | 10 |  |  |  |  |  |  |  |  |
